# Minimally invasive video-assisted thyroidectomy versus conventional thyroidectomy: A single-blinded, randomized controlled clinical trial

**DOI:** 10.4103/0972-9941.59307

**Published:** 2009

**Authors:** Gouda M El-Labban

**Affiliations:** Department of Surgery, Faculty of Medicine, Suez Canal University, Ismailia, Egypt

**Keywords:** Thyroid surgery, minimally invasive

## Abstract

We aimed to test the hypothesis that Minimally Invasive Video-assisted Thyroidectomy (MIVAT) affords comparable safety and efficacy as to the open conventional surgery, when dealing with patients with unilateral thyroid nodules or follicular lesions, in terms of cosmetic results, intraoperative and postoperative complications, postoperative pain and hospital stay.

**MATERIALS AND METHODS:**

This was a single-blinded randomised controlled trial comparing the MIVAT with conventional thyroidectomy. The primary endpoints of the study were measurement of postoperative pain after 24 and 48 hours from operation and self-rated patient satisfaction with cosmetic outcome three months postoperatively. The secondary outcome measures were operative time, incidence of temporary and permanent recurrent laryngeal nerve injury, postoperative haematoma formation, length of incision, and duration of hospital stay.

**RESULTS:**

Operative time was significantly less with open thyroidectomy than with MIVAT, while MIVAT was associated with less pain 24 hours postoperatively. Blood loss did not reach significance between procedures. Comparisons between the two procedures with regard to pain scores after 24 and 48 hours, respectively, depicted statistically significant differences in favour of the MIVAT after 24 hours. MIVAT was associated with less scarring and more satisfactory cosmetic results. There were statistically no significant differences between both procedures for the presence of transient recurrent laryngeal nerve palsy and hypoparathyroidism.

**CONCLUSIONS:**

MIVAT is a safe procedure that produces outcomes, in view of short-term adverse events, similar to those of open thyroidectomy, and is superior in terms of immediate postoperative pain and cosmetic results.

## INTRODUCTION

Minimally invasive surgery for the neck is one of the newest and most interesting applications. Many reports exist on the use of this technique in thyroid surgery, particularly with regard to eliminating the unattractive scars sometimes caused by conventional surgery.[1–11] Minimal-access thyroid surgery was conceived primarily in Europe and Asia. A number of groups[11–17] have made pioneering contributions to this field. While a variety of minimally invasive approaches have been endorsed, the technique most widely practiced in North America is minimally invasive video-assisted thyroidectomy (MIVAT), as originally described by Miccoli *et al*.[12] As with many new surgical techniques, adoption of MIVAT in the United States has been slow and somewhat deliberate. Increasingly, however, high-volume thyroid surgical centers have embraced this approach, and modest-sized case series have been published, detailing their experiences.[18–19] A more comprehensive reflection of the North American experience with MIVAT, shows that consolidated data have been compiled prospectively at four academic medical centers, paying specific attention to the safety and feasibility of this approach.[20] Several surgeons have reported their experiences with minimally invasive and video-assisted surgery of the neck.[2,12,13,21–40] Although all these evidence-based data reported short- and long-term outcome data after endoscopic resections for different thyroid diseases, and clearly showed advantages in comparison with the traditional procedures, mini-invasive thyroid surgery has not been accepted yet.[13,19,41–46] One of the reasons for this initial refusal is partly due to the technical difficulty of endoscopic resection requiring adequate training both in open and endoscopic procedures before safely performing gland resection.[46]

Minimally invasive video-assisted thyroidectomy (MIVAT) has the potential to offer similar advantages over conventional thyroidectomy. However, almost a decade after the early descriptions of endoscopic thyroidectomy, MIVAT remains in the early phase of its evolution, with a variety of techniques practiced in a relatively small number of specialist centers internationally.[47] While the feasibility of MIVAT approaches has been well documented, few studies have observed these techniques in the setting of a randomised trial. Minimally invasive approaches have demonstrated some advantages in terms of cosmetic and pain outcomes.[13,42] While this approach appears anecdotally to have benefits over conventional thyroidectomy, a randomised clinical trial is needed to avoid the selection bias, which is inherent in retrospective studies and surgical case series.[48]

The aim of this study was to compare the outcomes of MIVAT with conventional surgery in patients presenting with unilateral thyroid nodules or follicular lesions.

## MATERIALS AND METHODS

### Study design

A single-blinded, randomised clinical trial comparing MIVAT with conventional hemithyroidectomy was undertaken within the Suez Canal University Hospital. The trial was approved by the Faculty of Medicine, Suez Canal University Research Ethics Committee, and written informed consents were obtained from all participants prior to entry into the trial. The study population included those patients with unilateral thyroid nodules or follicular lesions requiring hemithyroidectomy for further histological diagnosis. Patients with small solitary toxic thyroid nodules were also eligible for participation. Patients were considered for randomisation if they had unilateral nodular disease, with a maximum nodule diameter of less than or equal to 3.0 cm and were able to give informed consent. The participants were considered ineligible if preoperative fine needle cytology showed thyroid carcinoma, if the nodule diameter was greater than 3.0 cm, active thyroiditis was evident, or there was a history of previous neck surgery or head and neck irradiation.

### Operative technique

Patients were randomised to undergo diagnostic hemithyroidectomy by either MIVAT or the conventional method. All the patients were blinded to the allocated procedure preoperatively. The procedure was performed by the same surgeon, and surgeon was aware of the procedure type at the time of randomisation. All patients underwent preoperative fiberoptic laryngoscopy to assess vocal cord movement. Both procedures were performed by a standardised technique. All patients had local infiltration of subcutaneous tissues beneath the incision with 5 ml of Marcaine, 0.5% with adrenaline.

The technique for MIVAT has been described previously by Miccoli *et al*.[13] The gasless video-assisted thyroid surgery was used. The patients were operated under general endotracheal anesthesia.

The patient was placed in a supine position and the neck was not hyperextended. Depending on the nodule size, a 2 cm or 2.5 cm horizontal skin incision was made 2 cm above the clavicle. An upper flap was created by subplatysmal dissection and elevated to create a tent-like working space, which provided a comfortable space for simultaneous insertion of a 3.3-mm 0° laparoscope and instruments through the same skin incision. With endoscopic assistance, subplastysmal dissection was carefully performed to avoid bleeding. The cervical linea alba was divided longitudinally as far up as the thyroid cartilage. The overlying strap muscles were dissected off the thyroid. The strap muscles on the affected side were retracted using an Army-Navy retractor to expose the thyroid and hold open the dissection space. A 10 Fr. suction catheter was attached to the scope for continuous suction of warm air in the wound, to prevent blurry scope optics. The middle thyroid vein or the small veins between the jugular vein and thyroid were divided with the help of a harmonic scalpel. An Allis tissue forceps was applied to the upper portion of the thyroid, allowing a downward and lateral traction of the thyroid. The avascular space between the upper pole of the thyroid and the cricothyroid muscle was opened to identify the external branch of the superior laryngeal nerve. The superior thyroid vessels were selectively isolated and divided using the harmonic scalpel. Subsequent to dividing the superior thyroid vessels, the upper portion of the thyroid was gently extracted from the incision using an Allis forceps. Gentle traction over the thyroid enabled the gland to be extracted without rupture. Then the inferior thyroid artery was exposed, and the parathyroid glands and recurrent laryngeal nerve were identified clearly. The inferior thyroid artery was ligated and not divided on the thyroid capsule, distal to its supply of the parathyroid glands. The thyroid was freed from the trachea by ligating the small vessels and dissecting the ligament of Berry. The isthmus was then dissected from the trachea and divided using the harmonic scalpel. The specimen excised was extracted from the wound and a small suction drain was left inside. The wound was closed with absorbable sutures.

Conventional hemithyroidectomy was performed by utilising a 5-6 cm Kocher incision and division of the ipsilateral strap muscles. After this exposure, the operative technique mirrored that used in the MIVAT approach. A standard dressing was applied for both MIVAT and conventional cases, with adhesive surgical tape placed horizontally across the neck. Patients were observed in the 24-hour ward and discharge was planned for the morning of the following day.

### Outcome measures

The primary endpoints of the study were measurement of postoperative pain after 24 and 48 hours of the operation and self-rated patient satisfaction with the cosmetic outcome, three months postoperatively. Postoperative pain scores were measured using a 10-point visual analogue scale (VAS) postoperatively. A higher numeric pain score represented more severe pain. Satisfaction with cosmetic outcome was measured at the follow-up using a 10-point VAS.

The secondary outcome measures were operative time, incidence of temporary and permanent recurrent laryngeal nerve injury, postoperative haematoma formation, length of incision, and duration of hospital stay.

The operative time was measured from initiation of the incision to conclusion of the skin closure, to the nearest minute. Recurrent laryngeal nerve function was assessed with preoperative and postoperative fiberoptic laryngoscopy. Postoperative haematoma was considered significant if it required return to the operating room (OR) for evacuation. Incision length was measured to the closest millimeter at the final follow up.

## RESULTS

### Patient characteristics

The clinical characteristics of the patients in the two groups were similar. There was a predominance of females in both groups, and the mean nodule size was equivalent between the groups, without any significant difference. The clinical characteristics are summarised in Table 1.

**Table 1:** Clinical characteristics of both studied groups

Clinical characteristics	MIVAT group (n = 38)	Conventional group (n = 38)	*P* value
Age (years)			
Mean ± SD	40 ± 17	42 ± 19	0.63
Gender			
Male/female (%)	11/27 (28.9/71.1)	10/28 (26.3/73.7)	0.70
Site of nodules			
Right lobe (%)	15 (39.5)	18 (47.4)	0.36
Left lobe (%)	13 (34.2)	15 (39.5)	
Isthmus (%)	10 (26.3)	5 (13.1)	
Nodule size by ultrasound (centimeters)			
Mean ± SD	2.7 ± 0.7	2.9 ± 0.3	0.11

### Surgical treatment

No patients in the MIVAT group required conversion to conventional surgery. The operative time as measured from the initiation of skin incision to the conclusion of subcuticular closure was greater for the MIVAT cases when compared with the conventional (*P* < 0.0001) cases. On an average, the MIVATS procedure had an operative time that was 16 minutes greater in duration than the conventional procedure. There were no significant differences in the estimated intraoperative blood loss or length of hospital stay. There were two patients who developed temporary recurrent laryngeal nerve paralysis in the MIVAT group and one in the conventional group. There was only one patient with permanent recurrent laryngeal nerve injury in the MIVAT group. No patient required return to the operating room for evacuation of haematoma. The operative details and complication rates are summarised in Table 2.

**Table 2:** Details of surgical treatment in both studied groups

Surgical details	MIVAT group (n = 38)	Conventional group (n = 38)	*P* value
Duration of procedure (minutes)			
Mean ± SD	62 ± 21	46 ± 5	< 0.0001**
Estimated blood loss (milliliter)			
Intraoperative Mean ± SD	39 ± 13.3	36.0 ± 19.5	0.44
Postoperative Mean ± SD	15 ± 2.5	14.2 ± 1.7	0.11
Duration of hospital stay (days)			
Mean ± SD	1.2 ± 0.4	1.04 ± 0.5	0.13
Recurrent laryngeal nerve dysfunction			
Temporary injury (%)	2 (5.3)	1 (2.6)	0.88
Permanent injury (%)	1 (2.6)	0	0.99
Haematoma			
Significant requiring return to OR	0	0	1.00
Insignificant	0	0	1.00
Wound infections			
No. (%)	2 (5.3)	2 (5.3)	1.00
Hypoparathyroidism		
No. (%)	2 (5.3)	2 (5.3)	1.00

### Outcome measures

Pain scores as measured on the 10-point VAS were significantly less in the MIVAT group after the first postoperative day when compared with the conventional group. The mean pain score after day one was 2.6 for the MIVAT group and 3.4 for the conventional group. There was no statistically significant difference in pain scores when measured 48 hours postoperatively (*P* > 0.05). The mean doses of intramuscular diclofenac sodium given after operation were significantly lower in the MIVAT group (40 mg) when compared with the conventional group (66 mg) (*P* < 0.0001). Three months postoperatively, participants in the MIVAT group reported a significantly greater satisfaction with the cosmetic outcome of their procedure compared to the conventional group. The mean satisfaction rating for the MIVAT group was 9.1 versus 4.9 for the conventional group on a scale of 1 to 10, with 10 representing the best possible outcome. In the MIVAT group, there was a significantly smaller incision length compared to the conventional group (3.2 ± 0.9 versus 5.4 ± 0.7 cm, respectively). The outcome data for pain scores and satisfaction with cosmetic appearance are summarised in Table 3.

**Table 3:** Outcomes after thyroidectomy treatment in both studied groups

Outcomes	MIVAT group (n = 38)	Conventional group (n = 38)	*P* value
VAS pain outcomes			
Pain score after 24 hours	2.6 ± 0.2	3.4 ± 0.6	< 0.0001**
Pain score after 48 hours	1.7 ± 0.1	1.8 ± 0.4	0.14
Dose of analgesic consumption postoperatively (diclofenac)			
Mean ± SD (mg)	40 ± 7.3	66 ± 12	< 0.0001**
Satisfaction with cosmetic results three months postoperatively			
Mean ± SD	9.1 ± 0.5	4.9 ± 0.6	< 0.0001**
Incision length (centimeters)			
Mean ± SD	3.2 ± 0.9	5.4 ± 0.7	< 0.0001**

## DISCUSSION

This study shows that in patients with small thyroid nodules the minimally invasive approach to thyroidectomy has some advantages over conventional thyroidectomy. The benefits of the MIVAT technique were demonstrated by less pain in the early postoperative period and superior cosmetic results at the three-month follow-up. The MIVAT approach represents a refinement in operative technique for thyroidectomy, which is applicable to small symptomatic nodules, toxic nodules, and follicular lesions, requiring further histological assessment.

The advantages of minimally invasive thyroidectomy have been demonstrated by other groups. As in this study, the major benefits center on reduction in pain and improvement in cosmetic results.[13,15,45,49–52] The majority of these studies have evaluated the MIVAT technique.

The operative time for MIVAT remains greater than that of conventional surgery, a finding which is common to a number of studies of minimally invasive approaches to the thyroid.[23,42,45] With greater experience, it is probable that operative times for MIVAT will decrease, particularly with the refinement of electrothermal vessel sealing devices, which have now become the preferred method for vessel control and dissection in open and minimally invasive thyroidectomy. This technology, in addition to the fact that MIVAT minimises the amount of unnecessary dissection required to expose the thyroid, will probably result in the decrease in operative time in the future. We hypothesise that the smaller skin incision and decreased area of dissection associated with MIVAT, results in less disruption of the cutaneous nerve supply, thus translating to less postoperative pain. To avoid the potential problem of information bias influencing the reporting of pain and cosmetic scores, we blinded patients preoperatively. Postoperatively, there is the potential for bias in reporting of pain scores from the MIVAT group; however, the combined reduction after day one pain scores and analgesic requirement suggests that the improvement effect is real. Similar benefits in terms of pain reduction have been reported in other series.[45,48,53]

## CONCLUSION

MIVAT is a safe procedure that produces outcomes; in view of short-term adverse events, similar to those of open thyroidectomy, it needs a longer operative time to be accomplished, but is superior in terms of immediate postoperative pain and cosmetic results.
